# Antitumor Properties of Matrikines of Different Origins: Prospects and Problems of Their Application

**DOI:** 10.3390/ijms24119502

**Published:** 2023-05-30

**Authors:** Aleksandr Popov, Emma Kozlovskaya, Tatyana Rutckova, Olga Styshova, Aleksey Vakhrushev, Elena Kupera, Ludmila Tekutyeva

**Affiliations:** 1 G.B. Elyakov Pacific Institute of Bioorganic Chemistry, Far Eastern Branch of the Russian Academy of Science, 159 Prospect 100-Letiya Vladivostoka, Vladivostok 690022, Russia; tanya1119@yandex.ru (T.R.); krivoshapkoon@mail.ru (O.S.); aivahr@mail.ru (A.V.); nkupera@gmail.ru (E.K.); 2 Department of Bioeconomy and Food Security, School of Economics and Management, Far Eastern Federal University, Vladivostok 690922, Russia; lat7777@mail.ru; 3 ARNIKA, Territory of PDA Nadezhdinskaya, Volno-Nadezhdinskoye 692481, Russia

**Keywords:** matrikines (MKs), collagen, antitumor activity, matrix metalloproteinases (MMPs), echinoderms

## Abstract

Matrikines (MKs) can be a rich source of functional nutrition components and additional therapy, thereby contributing to human health care and reducing the risk of developing serious diseases, including cancer. Currently, functionally active MKs as products of enzymatic transformation by matrix metalloproteinases (MMPs) are used for various biomedical purposes. Due to the absence of toxic side effects, low species specificity, relatively small size, and presence of various targets at the cell membranes, MKs often exhibit antitumor properties and, therefore, are promising agents for antitumor combination therapy. This review summarizes and analyzes the current data on the antitumor activity of MKs of different origins, discusses the problems and prospects for their therapeutic use, and evaluates the experimental results of studying the antitumor properties of MKs from different echinoderm species generated with the help of a complex of proteolytic enzymes from red king crab *Paralithodes camtschatica.* Special attention is paid to the analysis of possible mechanisms of the antitumor action of various functionally active MKs, products of the enzymatic activity of various MMPs, and the existing problems for their use in antitumor therapy.

## 1. Introduction

Peptide therapy with matrikines (MKs), biologically active compounds produced via partial proteolysis of collagen proteins and glycosaminoglycans of the extracellular matrix (ECM), has become increasingly popular on the pharmaceutical market. This is because they act as endogenous regulators of many physiological and pathological processes in the body. As a result, more than 60 peptide drugs approved by FDA (Food and Drug Administration, USA) are already used in practical medicine, and more than 600 have undergone clinical and preclinical trials. Accordingly, there is an increasing need for strategies to improve the properties of peptides, such as a longer half-life, higher bioavailability and increased efficiency [1,2].

The main advantage of regulatory MKs over chemical drugs is that being analogs of endogenous compounds, they rarely cause side reactions and exhibit a positive therapeutic effect in relatively small doses. Their activity is determined by the composition and amino acid sequence. The size of active peptides can vary from three to twenty amino acid residues. Due to their endogenous origin, low species specificity, relatively small size and the presence of various targets on the cell membranes, MKs often exhibit multifunctional properties and act as promising agents for targeted therapy of various pathologies, including tumor diseases [3,4,5]. However, despite some progress in the study of the antitumor properties of MKs, the arsenal of truly effective pharmacological agents based on them is extremely small.

A few years ago, the name “matrikines” was coined to refer to peptide products of partial proteolysis of ECM macromolecules capable of regulating cellular activity [6,7]. However, recent data indicate that some of these peptides may modulate proliferation, migration or apoptosis and play an important role in controlling tumor progression. Different modules of proteins that make up ECM macromolecules present new signals to cells in contact with them, capable of activating various intracellular signaling pathways and thus modulating numerous cellular functions. Taken together, it appears that many MKs are able to modulate the growth or invasive properties of tumor cells. In addition to its architectural role, ECM should now be seen as an integrated and dynamic system in which modules constitute a large array of signals to the surrounding cells. MKs produced by partial proteolysis of ECM macromolecules constitute a new family of messengers that control cell activity. Violations in the regulation of this system are often involved in modulating the growth of certain tumors in the connective tissues [5,6,7].

In connection with the above considerations, this review presents a comprehensive analysis of novel data on the antitumor activity of MKs obtained from various sources using endogenous and exogenous proteolytic enzymes. Possible mechanisms of action of these MKs, problems and prospects for the development of new antitumor agents are discussed. An important part of the review is focused on the studies of the antitumor activity of MKs from various marine echinoderm species obtained by proteolysis using original technology.

## 2. Sources of Matrikines

MKs originate from the fragmentation of ECM proteins of different compositions and structures, which regulate cellular activities by interacting with specific receptors. The structure and function of ECM change dramatically in the course of solid cancer development. Therefore, features of the structure and function of various sources of MKs are of great interest.

### 2.1. Collagen-Derived MKs

Collagen is the most abundant protein in mammals and accounts for over 90% of the total ECM proteins. Collagen fibers provide tensile strength to the ECM and scaffold for cell-to-cell communication. Twenty-eight different collagens containing 46 distinct polypeptide chains have been identified in vertebrates. Collagen polypeptides possess a primary structural Gly-Pro-X repeat (with X often being another proline) responsible for the characteristic right-handed helix secondary structure. The quaternary structure of collagen is a triple helix that contains two α_1_ chains and one α_2_ chain. Interstrand hydrogen bonding between proline and hydroxyproline hides the abundant glycine residues (at least one-third of the protein residues) within this trimeric collagen molecule, making them largely inaccessible in solution and thus protected from enzymatic degradation. This triple-helix strand can then be further assembled into either nonfibrillar collagen (type IV) or fibrillar collagen (types I, II, III, and V), with each fibril containing many collagen molecules [8,9,10,11,12,13]. Protection of the core glycine residues from proteolysis is important to maintain the structural integrity of collagen and to escape many inherited disorders of ECM stability [14].

Numerous studies have demonstrated the presence of strong regulatory potential in almost all collagen proteins and ECM peptidoglycans, which make up 25% of the mass of all extracellular proteins [8,9,10]. At the same time, it is important to understand how these MK precursors interact with the cell membrane receptors, regulating numerous signaling pathways both under normal conditions and in various pathologies, including tumorigenesis [11].

**Figure 1:**
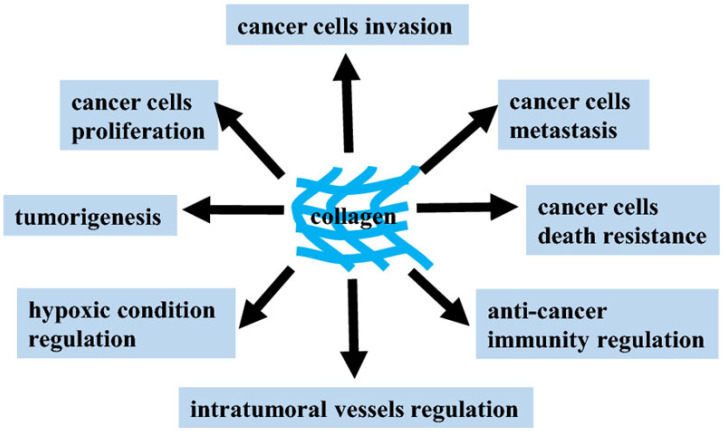
Potential contribution of collagens to the regulation and development of the tumor process [13].

It is important to emphasize that even minor changes in the content of collagens and proteoglycans in the ECM of cancer cells ultimately make a significant contribution to their resistance to therapeutic agents and tumor recurrence. Unlike normal epithelial cells, cancer cells do not need to be connected to the ECM in order to survive and proliferate. Due to the resistance to anoikis (a special case of apoptotic cell death) and without proper contact with the ECM, cancer cells can migrate throughout the body and metastasize. This is the reason for targeting pathogenic switches in integrins, tumor antigens, growth factors, and metabolic and signaling pathways acquired by the tumor during malignancy with many potential anticancer MKs. These MKs are in various phases of preclinical and clinical trials. The combinatorial approaches of modern chemotherapy can maximize therapeutic efficacy and minimize the activation of alternative signaling pathways that, on the contrary, may contribute to drug resistance. In this regard, a comprehensive analysis of the mechanisms of action of potential drugs and lead compounds among MKs can provide a rational basis for clinical applications of the respective agents for patients with distinct drug sensitivity profiles [10,11,12]. The pathophysiological functions of collagen in various types of cancer illustrate its dual role and pinpoint therapeutic strategies that can be used in clinical practice (Figure 1) [13].

The therapeutic use of a mimetic 20-amino acid peptide derived from type IV collagen has recently been studied in the treatment of breast cancer. The peptide decreased proliferation, adhesion, and migration of endothelial and tumor cells in vitro. In addition, there was a 75% inhibition of triple-negative human cancer cell MDA-MB-231 xenograft growth relative to control when the peptide was administered intraperitoneally for 27 days at 10 mg/kg. The treatment also resulted in an increase in caspase-3 activity and a reduction of microvascular density. The multiple modes of action of this peptide, both anti-angiogenic and anti-tumorigenic, make it a viable candidate as a therapeutic agent for monotherapy or in combination with other compounds [15].

### 2.2. Elastin-Derived MKs

Elastin is a polymer of tropoelastin that consists of lysine- and alanine-rich domains along with hydrophobic domains that are rich in glycine, valine, and proline repeats. In addition to compromising the structural integrity, proteolytic degradation of elastin enhances an ongoing pulmonary injury by liberating matrikines. Elastin fragments under 50 kDa were capable of potently inducing monocyte chemotaxis. Elastin hexamer VGVAPG repeat was found to be chemotactic to both monocytes and fibroblasts. Further studies demonstrated that this peptide upregulated various MMPs in endothelial cells, fibroblasts, and tumor cells. The conformation of the VGVAPG peptide is critical to its biological function, and the S-Gal receptor (also known as elastin-binding protein [EBP]) is thought to be the primary elastin receptor for this fragment. A variety of proteases degrade elastin, and their tight regulation is critical for the maintenance of health. Elastin-derived peptides are likely elicited by the elastases, macrophage metalloelastase (MMP12) and neutrophil elastase. The destruction of elastin promotes the development and progression of pathological conditions, including chronic obstructive pulmonary disease, atherosclerosis, vascular aneurysms, and cancer [14]. For example, in lung and colon cancer, the degradation of the matrix and fragmentation of elastin was found mainly to occur at the invasive front, and the expression levels of MMP also correlated to the metastatic potential of these cancers [16].

### 2.3. Hyaluronan-Derived MKs

Hyaluronan (HA) is an anionic glycosaminoglycan found throughout the ECM. HA comprises a large constituent of the basement membrane in solid organs. It is synthesized as a large polysaccharide repeat composed of N-acetyl-d-glycosamine and d-glucuronic acid, widely present in soft tissues. There are three HA synthase isoforms (HAS1–3) and six human hyaluronidase enzymes that degrade HA by hydrolysis of saccharide linkages. Exogenous hyaluronidases produced by bacteria or concentrated in snake and scorpion venom are pathologic factors that mediate tissue invasion and necrosis. The molecular weight of HA is a primary determinant of its function as a matrikine (MK). Like collagen and elastin, the common high-molecular-weight (>500 kDa) HA is important for the tissue’s structural characteristics. The low-molecular-weight (<100 kDa) HA isoforms can ligate receptors to mediate cellular signaling activity. Thus, the availability of tissue-active HA is regulated by the degree of HA hydrolysis or synthesis. Among HA’s effects, the induction of proteases or other matrix lytic enzymes is particularly relevant to its pathologic role. Low–molecular-weight HA induces transcriptional upregulation and release of active forms of MMP12 (which cleaves elastin) and MMP9 (which cleaves collagen), each of which can facilitate further MKs generation. HA activity impacts multiple aspects of cell migration, invasion, angiogenesis, and cellular differentiation [14]. Syndecans are transmembrane proteoglycans with heparan and chondroitin sulfate chains attached to their extracellular domain. They may also exist as soluble extracellular domains. Similar to many proteoglycans, they interact with a multitude of ligands, such as growth factors, adhesion receptors, proteinases, cytokines, chemokines and other ECM proteins to initiate downstream signaling responsible for proliferation, adhesion, angiogenesis, and inflammation. Elevated levels of syndecan expression in cancer can correlate with poor outcomes: Syndecan-1 in breast cancer and Syndecan-2 in colorectal cancer are highly associated with metastasis. HA binds to the cluster of differentiation 44 protein (CD44), a transmembrane receptor that participates in many physiological and pathological processes by activating key signaling cascades. Ligation of CD44 initiates the expression of genes related to tumor growth, proliferation, and survival. CD44 ligation with HA induces cytoskeletal rearrangements and membrane ruffling that leads to active cell migration. Further, CD44 serves as a marker for several types of stem cells [16].

### 2.4. Laminin-Derived MKs

Laminins are large-molecular-weight heterotrimeric glycoproteins that are designated with three number codes that correspond to their constituent α, β, and γ chains (e.g., laminin 111 or 543). These proteins are found in large quantities in tissue basement membranes, where the C-terminus of the laminin α subunit interacts with basolateral cellular plasma membranes, and the N-termini of all three subunits assemble through coiled-coil domains and project into the basement membrane to interact with other ECM components such as collagen or HA. These glycoproteins transduce both biochemical and mechanical signals between ECM and cells to influence cell survival, differentiation, and migration. Laminins are degraded by serine protease and metalloprotease family members. Laminin 332 (also known as laminin 5) is a component of multiple basement membranes and is implicated in hemidesmosome formation. The cleavage of the γ_2_ chain of laminin 332 by multiple MMPs (MMP3, -12, -13, -14, and -20) creates biologically active peptides that bind and ligate EGFR to mediate mitogenic epithelial activation and cell migration. MMP14-dependent γ_2_ cleavage in the lung generates fragments with EGF mimetic activity that induces regenerative alveologenesis. Apart from metalloprotease activity, the serine protease neutrophil elastase has been shown to cleave all three chains of laminin 332, resulting in global neutrophil chemotaxis. More specifically, the neutrophil elastase-mediated cleavage of the γ_2_ chain releases the γ_2_ peptide 597–618. This peptide neighbors the MMP2 cleavage site and is chemotactic for polymorphonuclear leukocytes in vitro, though the specific receptor for this peptide remains uncharacterized [14].

Of the synthetic MKs, peptide PCK3145 corresponding to amino acids 31–45 of prostate secretory protein 94 can reduce experimental skeletal metastases and prostate tumor growth. These anti-metastatic and anti-tumoral effects of PCK3145 are partially explained by the in vivo and in vitro decrease in matrix metalloproteinase (MMP)-9 extracellular levels through as yet unidentified molecular mechanisms. It can be assumed that PCK3145 rapidly triggers intracellular signaling through cell surface laminin receptors. This leads to decreased HuR expression and subsequent destabilization of MMP-9 transcripts. HuR protein is a nucleocytoplasmic protein that plays an important role in the regulation of mRNA stability. Dysregulation of its expression has been linked to carcinogenesis. This is the first molecular evidence demonstrating the intracellular signaling and anti-metastatic mechanism of action of PCK3145 that leads to the inhibition of MMP-9 secretion [16,17].

Thus, a complex ensemble of MKs from various sources modulate processes vital for normal and cancer cells, such as proliferation, migration, invasion, autophagy, and angiogenesis, managing tumor aggressiveness and metastatic potential of various malignant neoplasms. In the future, it is necessary to solve the complex problem of managing tumor processes and directing their development toward normalization using MKs and various new therapeutic approaches.

## 3. The Role of Various MPPs in Cancer Development

MMPs are a large family of Ca^2+^-dependent Zn^2+^-containing endopeptidases, which are responsible for the tissue remodeling and degradation of the ECM, including collagens, elastins, gelatin, matrix glycoproteins, and proteoglycan [18]. MMPs are secreted by various cells (fibroblasts, macrophages, smooth muscle cells of the vascular wall, neutrophils, chondrocytes, osteoblasts, keratocytes, etc.) and hydrolyze all components of the extracellular matrix (EM): all collagens and procollagens, proteoglycans, elastin, fibronectin, laminin, as well as adhesive and other connective tissue proteins. Under physiological conditions, there is a balance between the synthesis and breakdown of collagen, which prevents the process of tumor malignancy. Any change in the structure of the ECM means a violation of the stable balance between the rates of synthesis and degradation of its proteins. The degradation of ECM components is carried out by endogenous matrix MMPs. These enzymes play a crucial role in the development of such physiological processes as tissue remodeling, migration, adhesion, cell differentiation and proliferation. It is now known that an increase in MMP activity does not necessarily imply the promotion of tumor growth or metastasis. Moreover, some MMPs have been shown to play a protective role in cancer. It is important to note that the ECM changes strongly in tumors, and these changes can be both protumor and antitumor in nature. There is evidence that MMP-1, -3, -7, -9, -14, -16 and -19 can degrade and regulate endothelial vascular growth factor (VEGF) bioavailability and vascularization in cancer. Exposure of ECM components such as collagen IV, XVIII, perlecan, and heparan sulfate proteoglycan 2 (HSPG2) by various MMPs (-1, -2, -3, -9 or -13) can initiate the production of anti-angiogenic products, such as tumstatin, endostatin, angiostatin and endorepellin [19].

Overexpression and dysregulation of MMPs are often associated with various neoplastic diseases. Therefore, the regulation and inhibition of MMPs is an important therapeutic approach to combat these pathologies, and MMP inhibitors can be used as antitumor agents. However, to date, more than 50 different MMP inhibitors have been found to be ineffective in clinical trials [20,21]. Nevertheless, further research in this direction with the aim of developing new selective MMP inhibitors in the fight against cancer remains relevant. The initial concept of the involvement of MMPs in the development of malignancies was that inhibition of their proteolytic activity reduces ECM remodeling and prevents cell invasion and cancer metastasis [22]. However, often things are much more complicated. For example, increased expression and activity of MMP-8 are associated with good outcomes in oral squamous cell carcinoma and skin cancer but poor outcomes in ovarian, digestive tract, and hepatocellular cancer [23].

Previous generation MMP inhibitors have been developed and tested based only on the extracellular role of MMPs. These inhibitors were non-selective and have not been clinically tested in cancer therapy, mainly due to therapeutic dose-limiting toxicity. Collagen peptide-drug conjugates represent the current generation of targeted therapeutics, like other well-known antibody-drug conjugates. The main advantages of these drugs are increased cell permeability and relatively high selectivity. BT1718 is a drug designed to target and inhibit the function of membrane metalloproteinase type 1 (MT1-MMP) and MMP-2. This drug is usually well tolerated during the course of antitumor therapy, and current data on the pharmacokinetics in blood plasma and tumors is consistent with the proposed preclinical mechanism for targeted delivery of the toxin to the tumor. MT1-MMP and MMP-2 are involved in the breakdown of proteins normally surrounding the cell; however, within cancer cells, these proteinases promote their growth and invasion. The content of MT1-MMP and MMP-2 in normal cells is usually low but can reach higher levels in cancer cells. A growing body of evidence shows that MT1-MMP and MMP-2 are present and active in different subcellular structures. This is important because the discovery of new roles for MT1-MMP and MMP-2 outside the ECM will be of great importance in anticancer therapy [24].

The first clinical trials of the hybrid drug BT1718 showed promising results. However, future studies are needed to identify new intracellular substrates and epigenetic functions of MT1-MMP and MMP-2 in cancer. For example, nuclear MT1-MMP and nuclear/nucleolar MMP-2 may be new therapeutic targets in metastatic cancers. This will help develop more specific inhibitors that will increase the therapeutic efficacy of their interventions in neoplastic diseases [25]. It should be noted that MMP-14 is also the driving force behind tissue destruction during cancer invasion and metastasis and has a significant effect on intercellular communication, regulating the activity of many plasma membrane-anchored and extracellular proteins. For this reason, MMP-14 is an important target for screening new selective inhibitors [26].

There is ample evidence that MMPs are strongly involved in tumor invasion and metastasis tumor growth processes. For example, the results of increased expression of individual MMPs in tumors and the association of specific MMPs with poor prognosis. Recent studies highlight and enhance the role of specific MMPs as key players in tumor growth processes [27].

The tumor microenvironment is formed by cells as well as ECM components and their complex interactions in and around a solid tumor mass. MMPs can degrade ECM components to release MKs. These bioactive peptides often regulate tumor progression and metastasis and can be used for diagnostic purposes. For example, the degradation of perlecan by MMP leads to the formation of several fragments, in particular, the angiostatic endorepellin. Subsequent proteolytic cleavage of endorepellin by proteases and cathepsin L releases a laminin G-like domain that binds the α2β1 integrin. In addition, plasma membrane-associated proteins, such as the G-protein-coupled adhesion B1 receptor and orphan G-protein-coupled receptor protein, can also be cleaved by MMP-14. As a result, such biologically active molecules with a matrikin-like function as angiogenesis-inhibiting vasculostatin-40 are formed. Conversely, MMP activity is regulated by MKs. For example, lamstatin (NC1), the C-terminal domain of the α5-chain of type IV collagen, inhibits tumor cell migration by suppressing both αvβ3 integrin and MMP-14 [28]. Lumican (a small, leucine-rich proteoglycan) binds to MMP-14, selectively inhibits protease activity, and prevents collagen degradation, limiting tumor invasion and progression [29].

Therefore, the study of the main functional mechanisms of the interaction of MKs with MMPs is extremely useful for the development of new selective antitumor agents, markers and treatments. Each MMP has important specific functions both in normal conditions and during tumor development. Given the established role of MMPs in cancer progression and metastasis, it is reasonable to continue to consider them as potential therapeutic targets in the development of cancer therapies.

## 4. Problems and Prospects for the Use of Various MKs as Anticancer Agents

**Figure 2:**
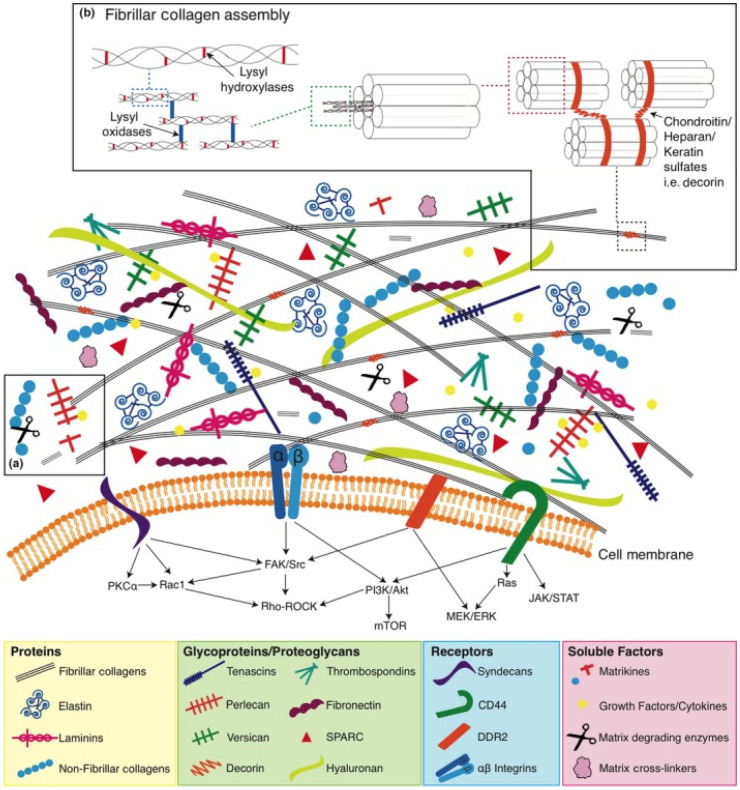
Simplified representation of the ECM, which is made up of many MK molecules that come together to form highly ordered supramolecular structures. Through cell surface receptors, MKs modulate a number of downstream intracellular signaling pathways that determine the functional activity of cells. (**a**) soluble MKs and matrix degrading enzymes; (**b**) fibrillar collagen assembly [33].

Our knowledge about the structure and functions of the ECM has expanded significantly over the past decades. There is evidence that ECM components (MKs) provide signals that affect the adhesion, migration, proliferation, survival and differentiation of various cell types due to the content of a large number of domain structures that become active after proteolytic cleavage. Moreover, these active fragments of ECM components can act as powerful inflammatory mediators for tissue damage. A growing body of evidence suggests that the ECM plays an important role in tumor development through changes in macromolecular components, degradation enzymes and tissue stiffness. These variations are controlled by cellular components in tumor tissue through the aberrant activation of signaling pathways and the interaction of ECM components with multiple surface receptors. Moreover, the deep regulatory network of ECM remodeling hinders effective antitumor therapy [30]. Therefore, it must be emphasized that the “normalization” of the ECM should be an essential part of the overall strategy for the treatment and prevention of cancer. Due to significant progress in understanding the role of ECM communications in cancer development, interest has increased in the development of new antitumor therapy based on MKs, the effect of which is aimed at selective inhibition of the tumor process [31,32]. Loss of tissue homeostasis and integrity of the ECM is regarded as one of the hallmarks of cancer and usually defines transient events during progression and metastasis. The vast majority of oncological research is focused on studying the pathological features of the behavior and functioning of the internal signaling pathways of tumor cells. When studying the antitumor activity of MK, the main task is to search for specific targets, the interaction with which makes it possible to effectively inhibit the development of tumor processes. The ECM and its fragments (MK) have a strong influence on cancer progression and metastasis and thus represent a vast unexplored repository of potential anticancer targets that we are just beginning to identify and use in antitumor therapy (Figure 2) [33].

New strategies for highly selective pharmacological influence on key ECM partners should include promising new MKs to develop effective, personalized therapeutic approaches to cancer treatment. The use of antitumor MKs is of great interest because (1) these molecules are small in size, (2) they exhibit good cellular diffusion and permeability, (3) they affect one or more specific molecular pathways involved in carcinogenesis, and (4) they are usually not genotoxic [34].

Relatively recently, the most well-known autophagic matrix modulators, including MKs, have been reviewed, and a modern understanding of the cellular pathways and signaling cascades that control autophagic function has been presented. The authors critically assessed how autophagic functions affect oncogenesis, emphasizing the complexity of these relationships, depending on the stage of development of these processes, as well as the features of the action of pro- and anti-autophagic modulators, which are often degradation products of the ECM. For example, various MKs can have both positive and negative effects on tumor growth and development [35].

As an important part of MKs, the various fragmented collagens and proteoglycans are complex molecules comprising a protein backbone with sulfated glycosaminoglycan chains of various chemical types covalently attached. Most proteoglycans are secreted or attached to the cell membrane. Their specialized structures, binding properties and biophysical attributes underlie a variety of biological roles that include modulation of tissue mechanics, cell adhesion, sequestration and controlled release of morphogens, growth factors, and cytokines. Proteolysis is normally required for molecular maturation of some collagens and proteoglycans, clearance of ECM proteoglycans during tissue remodeling, and formation of effector MKs. On the contrary, unregulated proteolysis of collagens and proteoglycans contributes to the emergence of various complex pathologies, including cancer [35].

Collagen XVIII, a collagen-heparan sulfate (HSPG) hybrid proteoglycan, has a dual function as a pro- and anti-angiogenic factor. Through heparan sulfate chains, this proteoglycan can stimulate angiogenic signaling by sequestering, protecting and concentrating HS-binding growth factors such as fibroblast growth factor, endothelial vascular growth factor and platelet growth factor. In addition, as a result of proteolytic processing of the HSPG C-terminal domain, these gene products can release powerful angiostatic fragments, such as endostatin and endorepellin, which act locally or remotely on the paracrine functions of growing endothelial cells [36].

Endostatin, a 20 kDa C-terminal fragment of collagen XVIII, was first identified as a potent inhibitor of angiogenesis. Recently, several studies have shown that endostatin also inhibits tumor lymphangiogenesis and lymphatic metastases [37]. In addition, recombinant human endostatin (Endostar) can inhibit the expression of vascular endothelial growth factor and, consequently, tumor angiogenesis and tumor metastasis [38,39].

Previous clinical trials have demonstrated a pronounced antitumor activity of Endostar in cancer patients. Several studies have shown encouraging results with Endostar in the treatment of sarcoma in combination with known chemotherapy drugs. It is suggested that combination therapy may improve the prognosis in patients with soft tissue sarcoma, especially those with undifferentiated polymorphic sarcoma [40]. Research over the past decade has shown that the effects of endostatin are complex and involve multiple mechanisms. However, the exact mechanism by which endostatin exerts its anti-angiogenic functions remains unclear [41].

Endorepellin, the C-terminal fragment of the heparan sulfate proteoglycan perlecan, influences various signaling pathways in endothelial cells by selectively binding to the vascular endothelial growth factor receptor 2 (VEGFR2). It is active at nanomolar concentrations and blocks endothelial cell adhesion to fibronectin and type I collagen without directly binding to either protein. Endothelial cells have a large number of endorepellin binding sites with high affinity [42]. In other words, endorepellin is a novel anti-angiogenic product that can slow tumor neovascularization and hence tumor growth in vivo. It activates the canonical stress signaling pathway, consisting of PERK kinase, eIF2α initiation factor, ATF4 and GADD45α effector proteins. In particular, endorepellin causes transient activation of VEGFR2, which in turn phosphorylates PERK at Thr980. Subsequently, PERK phosphorylates the α subunit of translation initiation factor eIF2α at Ser51, enhancing its downstream ATF4 and GADD45α. RNAi-mediated knockdown of PERK or eIF2α abolishes endorepellin-mediated upregulation of GADD45α, the final effector protein of this stress signaling cascade. These results reveal the mechanism by which the ECM peptide fragment endorepellin induces stress signaling in endothelial cells, leading to angiostasis [42].

Thus, endorepellin is a novel anti-angiogenic product that can slow tumor neovascularization and hence tumor growth in vivo. Type IV collagen is the main component of the basement membrane of blood vessels and is known as a fragment of the alpha2 chain with specific antiangiogenic properties [43]. In addition, the antitumor activities associated with the non-collagen domain 1 (NC1) of the alpha3 chain of type IV collagen have been characterized. This domain contains a C-terminal peptide fragment (amino acids 185–203) that inhibits melanoma cell proliferation [44].

Tumstatin, a non-collagen type IV collagen α3 chain, or α3 (IV) NC1, is a specific inhibitor of translation and angiogenesis in endothelial cells. It binds surface integrins to cells. When interacting with integrin αVβ3 in endothelial cells, tumstatin inhibits phosphorylation of focal adhesion kinase (FAK), which leads to suppression of the activation of Ca^2+^-dependent translation of the FAK/PI-3K/Akt/mTOR/eIF4E/4E-BP1 axis, activation of apoptosis and cell death. In addition, Tumstatin transdominantly inhibits αVβ3 integrin expression in cells and suppresses NF-κB transcription factor-mediated signaling under hypoxic conditions. This leads to inhibition of the expression of the COX-2/VEGF/bFGF axis and suppression of hypoxic tumor angiogenesis [45].

When determining the anti-angiogenic ability of tumstatin, it was shown to inhibit neovascularization in vitvo and suppress tumor growth of human renal cell carcinoma and prostate carcinoma in xenograft models in mice in vivo. The anti-angiogenic activity was localized at amino acids 54–132 using deletion mutagenesis. This antiangiogenic site is separate from the 185–203 amino acid site responsible for antitumor activity. At the same time, antitumor activity is not realized until this region of the peptide is exposed to truncation, which is not essential for the antiangiogenic activity of this domain [46].

Overall, these results clearly highlight both the unique antitumor properties of tumstatin and the potential use of these MKs to inhibit tumor growth. It is important to emphasize that other ECM fragments (arresten, canstatin, vastatin, restin, endostatin, and endorepellin) have completely different functions than their original full-length proteins [47]. The best-known autophagic matrix modulator perlecan is the important chondroitin sulfate proteoglycan (HSPG). Perlecan regulates a wide range of biological processes, not limited to angiogenesis, autophagy, endocytosis, cell adhesion, thrombosis, blood–brain barrier integrity and lipid catabolism. In contrast to its pro-autophagic C-terminal V domain of endorepellin, perlecan generally inhibits autophagy through activation of the mammalian target of rapamycin complex 1. Another small leucine-rich proteoglycan, lumican, regulates a variety of physiological and pathological processes, including fibrocyte differentiation, wound healing, glucose homeostasis, inflammation, cell migration and cancer [48].

Many papers have now critically assessed how autophagic functions influence tumorigenesis, highlighting the complexity and stage-dependent nature of this relationship in cancer. Pro- and anti-autophagic MK modulators derived from the ECM specifically affect the progression and inhibition of cancer [35,48]. In this regard, further in-depth study of the effect of MKs on cellular biochemical pathways and signaling cascades is necessary, as well as the determination of specific mechanisms for controlling autophagic function during tumor development since many very important unresolved issues remain. For example, given the role of autophagy in oncogenesis, at what stage do pro- and anti-autophagic MKs play a role in either suppressing or promoting tumor development? Do the subsequent effects of these autophagic modulators occur separately, or are they coordinated synergistically in the tumor microenvironment? Are some MKs more important in modulating disease-altering autophagy in certain cancers than others?

Thus, understanding the interaction of anti- and pro-autophagic MK molecules in aberrant ECM remodeling and its respective functions in oncogenesis should not be overlooked. In general, it can be concluded that further studies are needed to clearly understand the molecular mechanisms of the impact of MKs of different sizes, structures, and origins on the development of the tumor process.

## 5. Antitumor Activities of MKs from Echinoderms Obtained by Original Technology

In recent decades, various species of echinoderms, especially holothurians (holothurians), have gained popularity among researchers as an ideal source of collagens and proteoglycans. Most holothurians contain higher levels of these proteins and peptides and lower levels of lipids than other animal products, suggesting their potential benefits in terms of healthy, functional nutrition. The use of marine collagens is a hot topic in the field of tissue engineering and cosmetology. A distinctive feature of the physiology and biochemistry of holothurians is their amazing ability to regenerate lost tissues. It is believed that the regular use of holothurian (trepang) *Apostichopus japonicus* rejuvenates the body, which is often associated with the unique properties of mutable collagen tissues (MCTs) of holothurians, which, when life is threatened, make their body rigid and, if necessary, jelly-like. MCT echinoderms can represent an innovative source of collagen to develop collagen barrier membranes for Guided Tissue Regeneration, a technique for restoring bone tissue, originally developed for periodontal surgery and now widely used in dental implantology [49,50].

**Figure 3:**
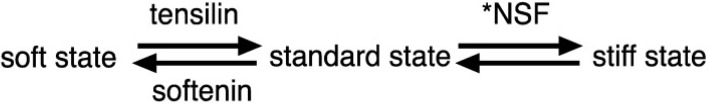
Three-state model of stiffness of the dermis of sea cucumbers. *NSF: novel stiffening facto [49].

Tensilin, a tissue stiffener, and softenin, a tissue softener, acting directly on the ECM, have been purified from the dermis of sea cucumbers, which is a typical catch connective tissue. Still, the molecular mechanism of the change in stiffness is not fully understood. Therefore, understanding the mechanisms of MCT quantitatively may have applications in the development of new types of mechanically tunable biomaterials (Figure 3) [49,50].

For this reason, the studies of the antitumor properties of echinoderm MKs obtained by enzymatic hydrolysis with a complex of proteolytic enzymes Collagenase KK from the hepatopancreas of the king crab *Paralithodes camtschatica* are of particular interest. Therefore, to generate MKs from the body of various Far Eastern echinoderm species, protocols were developed for isolating MKs from commercial species of sea holothurians, namely: trepang *Apostichopus japonicus* (MKT) and *Cucumaria japonica* (MKC), the widespread starfish *Patiria pectinifera* (MKS) and the spiny sea urchin *Strongylocentrotus nudus* (MKUn), as well as the endemic flat sea urchin *Scaphechinus mirabilis* (MKUm) [2,32,51].

**Figure 4:**
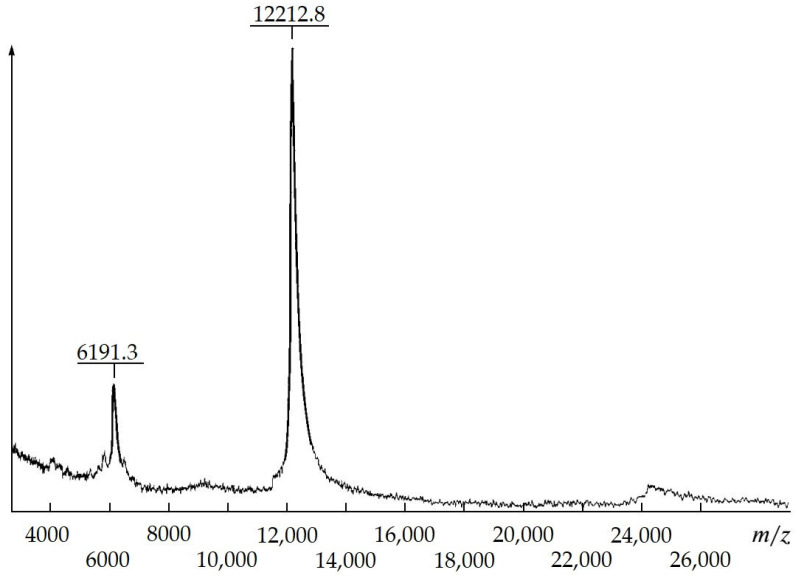
Chromato-mass spectrum of proteolytically modified collagen proteins from the holothurian *Apostichopus japonicus* (MKT). On the abscissa, ion mass to charge ratio; on the ordinate, relative intensity of ionic current [51].

Homogeneous MKs with various therapeutic effects by means of proteolytic modification, *A. japonicus* trepang collagens and proteoglycans, were obtained. According to MALDI mass spectrometry, the molecular weight of trepang peptides is about 6 and 12 kDa (Figure 4).

The end products of the enzymatic hydrolysis of echinoderm body proteins were freeze-dried MKs, highly soluble in water and biological fluids. Our study of the physicochemical properties of these products by various methods and the determination of the amino acid composition showed that the obtained collagen peptides are peptides of various sizes and compositions that preserve the spatial structure of native collagen [2,51,52,53]. Elemental and amino acid analysis of MKT, MKC and MKS suggests that they can be considered as typical collagen fragments containing 6% sulfated carbohydrate and/or amino acid residues. The MK compositions of all studied echinoderm species contain low-molecular-weight peptides (molecular weight > 2000 Da) and amino acids. The MKS mass spectrometric analysis data confirm the presence of medium-molecular-weight peptides—peptides 5961.2, 7124.8 and 8326.5 Da. The composition of MKC, according to mass spectrometry, contained a wide range of peptides by molecular weight. In this case, the presence of polypeptides with molecular weights of 24,534.9, 13,961.5, 11,537.7 and 8586.6 Da was found. No high-molecular-weight peptides were found during the enzymatic lysis of sea urchin collagen proteins MKUm and MKUn [2].

It should be noted that the data of mass spectrometric analysis also clearly demonstrates that the studied echinoderm MKs contain unique amino acid sequences that are not cleaved by trypsin-like proteinases and true collagenases. Thus, when analyzing an aqueous solution of a high-molecular-weight peptide (~22 kDa) from CPS by CD spectroscopy, it was found that high-molecular-weight peptides retain the spatial structure of collagen: a negative band of high ellipticity is present in the spectrum in the region of 210–220 nm. A comparative analysis of the amino acid composition of peptides isolated from various species of marine echinoderms indicates that the studied peptides of marine echinoderms are collagen fragments that differ in amino acid composition and differ significantly in this indicator from the composition of mollusk and fish peptides [2,51,52,53].

In a series of experiments to study the antitumor activity of MKT in vivo, its optimal therapeutic doses in relation to mouse models of solid and ascitic variants of Ehrlich’s tumor were determined. It has been shown that at doses from 10 to 40 mg/kg, MKT has a dose-dependent inhibitory effect only on the growth of solid tumors. The mean tumor weight in the control group was almost twice as high (*p* < 0.05) as in the experimental group of mice treated with test MKT at a dose of 20 mg/kg. The values of the mean tumor mass were 1252 ± 234 mg in the control group and 698 ± 125 mg in the experimental group. Cucumaria preparation MKC at a dose of 40 mg/kg also had a significant inhibitory effect on the growth of Ehrlich’s solid tumor: inhibition of tumor growth was 34.2 ± 4.8% compared to the untreated group (*p* < 0.05). In the case of a mouse model of the ascitic variant of Ehrlich’s tumor, MKT was less effective. The course of treatment, which included 10 consecutive injections of MKT one day after tumor inoculation, caused an increase in the average life expectancy of experimental animals by 29.4 ± 3.2% compared with the control group only at a dose of 40 mg/kg (*p* < 0.05). However, the MKC preparation, when used in a similar regimen with MKT at doses of 10 to 40 mg/kg, did not show activity [2,51,53].

Evaluation of the antitumor activity of MKS showed that in the range from 2.5 to 10 mg/kg, these peptides had a pronounced dose-dependent efficacy against a mouse model of a solid variant of Ehrlich’s tumor. The greatest antitumor effect was observed at 5 mg/kg (56.75 ± 17.65%) with five times daily treatment, starting the next day after tumor inoculation. Monotherapy with cyclophosphamide (CP) at a dose of 5 mg/kg (positive control) gave an antitumor effect of 40 ± 14.55%. The combined use of MKS and CP at a dose of 5 mg/kg showed that these compounds do not have a synergistic effect in this treatment regimen. It should be noted that at a dose of 2.5 mg/kg, MKS did not have significant antitumor activity (3.29 ± 17.74%). Apparently, the nature of MKS growth inhibition is similar to that of MKs of trepang and cucumaria [2,51,53].

It should be noted that in experiments on a solid rat model of carcinosarcoma, Walker 256 MKT, administered intraperitoneally and sequentially for 7 days at a dose of 20 mg/kg, significantly (*p* < 0.05) enhanced the antitumor and antimetastatic effects of the known cytostatic drug CP [54].

The results of the studies on the antitumor activity of MKT, MKC and MKS are in good agreement with the data of numerous studies of endostatin, which generally exhibits a weak inhibitory effect or is not active against ascitic tumors. This is probably due to the predominant antiangiogenic mechanism of action of these MKs on the growth of solid tumors [2,51,52]. It is known that endostatin induces apoptosis of endothelial cells, inhibits the growth of blood vessels feeding tumors and creates conditions for inhibition of tumor cell metastasis [37,38,39,55].

Thus, the effectiveness of the antitumor action of echinoderm MKs is determined by the structural features of proteoglycan and collagen fragments of different lengths, amino acid composition, and the presence of sulfated carbohydrate chains. Further therapeutic studies of the antitumor properties of MKs from echinoderms, especially holothurians, are supported by the absence of toxic side effects and stimulating action on the growth of experimental tumors in vivo. It should be emphasized that echinoderm MKs often exhibit therapeutic effects when used to treat other various pathologies [2,51,53].

## 6. Conclusions

**Figure 5:**
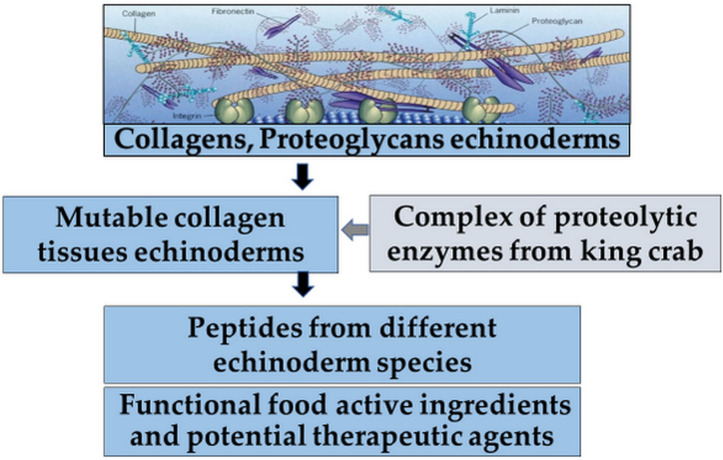
Prospects for the use of MKs from different species of echinoderms obtained by enzymatic hydrolysis.

Summarizing the data presented above, MKs exhibiting antitumor activity represent a growing alternative as chemotherapeutic agents but are still rarely represented in the development of new functional food ingredients and potential therapeutic agents (Figure 5) [2,51,53].

Although the ECM often forms the main component of the tumor microenvironment, the role of its MKs in cancer is poorly understood. The convergence of modern imaging techniques, ohmic technologies and advanced bioinformatics methods contributes to the development of modern research and quantification of the role of MKs from different types of organisms both in the development and treatment of cancer. In addition to the possible function of directly interfering with the signaling and biochemical pathways that cause tumor disease or initiating apoptosis, MKs can be used in the future as additional means of enhancing traditional antitumor chemotherapy and minimizing its toxic side effects. The antitumor properties of MKs from various sources, including marine echinoderms, noted in this review, allow us to consider them as possible effective components of complex drugs and functional foods. The results of a study of the antitumor properties of various echinoderm MKs obtained by biocatalytic conversion show that it, like endostatin, demonstrates antitumor activity but acts with varying degrees of effectiveness and can be recommended for further research.

Thus, a comprehensive study of the MKs’ structure–activity relationship in the future will provide a fundamental basis for understanding the mechanisms of their therapeutic effects. In addition, this will allow one to consider their individual representatives as important components of therapeutic agents in the future. Furthermore, the biotechnological potential of MKs from various echinoderm species, as components of MCTs, can become a source of new strategies for therapeutic manipulation of the mechanical and biochemical properties of cells and tissues of the body and the development of new composite materials for biomedical applications.

## Data Availability

Not applicable.
